# Evaluation of postoperative pain in infected root canals after using double antibiotic paste versus calcium hydroxide as intra-canal medication: A randomized controlled trial

**DOI:** 10.12688/f1000research.16820.1

**Published:** 2018-11-08

**Authors:** Sarah Samir Abouelenien, Salsabyl Mohamed Ibrahim, Olfat Gameel Shaker, Geraldine Mohamed Ahmed

**Affiliations:** 1 Department of Endodontics, Faculty of Oral and Dental Medicine, Cairo University, Cairo, 11553, Egypt; 2 Medical Biochemistry and Molecular Biology, Cairo University, Cairo, 11553, Egypt

**Keywords:** apical periodontitis, calcium hydroxide, antibiotic paste, intra-canal medication, postoperative pain

## Abstract

**Background:** Postoperative pain is defined as pain of any degree after initiation of endodontic treatment either intra-appointment or post-obturation and is considered an undesirable occurrence for both patient and dentist. It was suggested that bacterial injury is probably the major cause of pain. Intra-canal medicaments are widely used to kill any bacteria surviving after instrumentation and irrigation. The aim of this study was to assess the ability of double antibiotic paste versus calcium hydroxide used as intra-canal medication in reducing postoperative pain.

**Methods:** 36 patients with single rooted necrotic premolars with apical periodontitis were randomly assigned into two groups according to the intra-canal medication used: calcium hydroxide group (CH) and double antibiotic paste group (DAP). Preoperative pain was recorded using numerical rating scale. After isolation, access cavity was performed followed by chemico-mechanical preparation using rotary Race files with 2.5% sodium hypochlorite irrigation. Subsequently, intra-canal medication was placed and postoperative pain was recorded at 6, 12, 24 and 48 hours postoperatively.

**Results: **There was no statistically significant difference between both groups. Both groups resulted in an increase in median pain value from preoperative to 6 hours postoperative, followed by gradual decrease from 6 hours to 12, 24, 48 hours postoperatively with statistically significant difference. When comparing both groups, DAP group showed lower postoperative pain values than CH group at 12 and 24 hours, but this was not statistically significant.

**Conclusion:** The use of intra-canal medication in necrotic teeth with apical periodontitis was efficient in reducing postoperative pain regardless of type of intra-canal medication used.

**Trial registration:** PACTR201605001482394 (Date: 22 ^nd^ February 2016).

## Introduction

Pain is the main symptom in many medical and dental conditions that can significantly alter the person’s general functioning ^1^. Post-endodontic pain remains a serious problem facing the dental profession. It may occur as a result of several factors, including chemical, mechanical or bacterial irritation to the periapical tissues ^2^. It has been suggested that the presence of bacteria as a result of failure to properly disinfect the root canal is the major cause of pain ^3^. Many studies have found a direct relationship between root canal infection and the level of pain after endodontic treatment ^4^. Thus, endodontic treatment is primarily focused on utmost elimination of these bacteria. Antibacterial intra-canal medicaments have been advocated in efforts to kill any bacteria surviving after chemico-mechanical preparation.

Several intra-canal medications have been used, including calcium hydroxide which is the most widely used intra-canal medication. It is characterized by its initial bactericidal, bacteriostatic effect and its high pH ^5^. It has been suggested that calcium hydroxide has pain preventive characteristics due to its antibacterial action or tissue altering effects. Moreover, it controls inflammation and has repair potential ^6^. However, there is no clear evidence of its effect on post-endodontic pain ^7^. Other studies found that calcium hydroxide was not very effective in decreasing post-treatment pain when used alone ^8, 9^.

Double antibiotic paste is a mixture of metronidazole and ciprofloxacin, which is used as intra-canal medication for disinfection of necrotic teeth. It has been reported to be effective at reducing bacterial counts in the infected root canals ^10^. When commonly used medicaments fail in eliminating the symptoms, then antibiotic paste can be used for treatment of teeth with large periapical lesions ^11^.

The objectives of the present study were to test the null hypothesis of whether the use of double antibiotic paste will be more effective in reduction of pain than calcium hydroxide, or if this paste will have disadvantages and complaints more than the traditional method used.

## Methods

The article has been written in concordance with CONSORT guidelines ( Supplementary File 1).

### Study setting

The study design is a parallel randomized controlled trial with allocation ratio 1:1 and a Superiority Framework. The study was conducted in the duration between March 2016 and March 2017. The protocol of this study was approved by the Ethics Committee of the Faculty of Oral and Dental Medicine, Cairo University, Egypt (approval number 15918). The nature of this study, and associated risks were fully explained to the patients and informed consent forms were signed before initial treatment.

### Sample size

Based on a previous study by Ehrmann *et al*. (2003), a medium effect size of approximately 0.25 is expected. A total sample size of 24 patients will be sufficient to detect an effect size of 0.25, a power of 80%, and a significance level of 5%. This number has been increased to a total sample size of 28, to adjust for using nonparametric tests. The number is again increased to a total sample size of 36 (18 in each of the two groups) to allow for losses of around 25%. Sample size was calculated using G*Power program (University of Düsseldorf, Düsseldorf, Germany). Minimal clinical difference 3.5 and 4.5 at 24 hrs and 48 hrs respectively.

### Participants

In total, 36 participants were included in the study with maxillary or mandibular non-vital single rooted premolar teeth showing tenderness to percussion.

*Exclusion criteria:* pregnant women, patients who had received antibiotic treatment during the last 3 months, patients having more than one tooth requiring root canal treatment, teeth with periodontal probing depth >4mm, teeth that couldn't be isolated with a rubber dam, teeth with previous root canal treatment, or teeth with fluctuant facial swelling where emergency management should include incision and drainage.

Patients were randomly assigned into two groups (n=18/group) according to the intra-canal medication used, using a computer generated random number table at the Center of Evidence-based Dentistry (EBD), Cairo University. The assistant supervisor generated the random sequence and assigned the participants to the intervention or control groups.

Experimental group: double antibiotic paste (DAP); control group: calcium hydroxide paste (CH). All clinical procedures were performed by a single endodontist.

### Intervention

All patients accepted two-visit root canal treatment. Preoperative pain was recorded before any intervention.

*First visit:* Teeth were anaesthetized using nerve block or infiltration technique by local anesthesia (Articaine HCI 4% & Adrenaline 1:100,000) and properly isolated with rubber dam. After access cavity preparation, the working length was checked using apex locator and confirmed by a radiograph. The root canals were prepared using rotary Race files (Fkg Dentaire, Switzerland) along with 2.5% sodium hypochlorite irrigation. The canals were dried and filled with intra-canal medication plugged into the canal by using Lentulo spiral according to the randomly selected group (DAP group - combination of 500 mg ciprofloxacin and 500 mg metronidazole ground then mixed with saline to obtain creamy consistency; CH group). The access cavities were properly filled with glass ionomer to ensure proper sealing with no leakage of any oral fluids inside the root canal, which might disturb the action of the intra-canal medication.

Patients were instructed to record their postoperative pain level (see below) after 6, 12, 24 and 48 hours.

*Second visit:* After 7 days, all the patients returned the pain charts and the root canals were obturated using lateral condensation technique and resin-based root canal sealer.

### Outcomes

Primary outcome measure is postoperative pain.

A pain chart using numerical rating scale (NRS) was used to record the patients’ pain level. The NRS (0–10 scale) consists of a line anchored by two extremes “No pain” and “the worst pain”. Patients were asked to choose the mark that represented their level of pain from 0 to 10. Pain level was assigned as follow: 0, “no pain”; 1–3, “mild pain”; 4–6, “moderate pain”; 7-10, “severe pain”.

### Blinding

The study was double-blinded (the participants and the assessor). Participants did not know which group they were treated with; they chose a folded paper inside an opaque envelope which contained the numbers of each random sequence for both groups. The assessor, who assessed all outcome data, did not know which group the participants were related to.

### Statistical analysis

Data was analyzed using IBM SPSS 21 (SPSS Inc., Chicago, IL, USA). The level of pain was described as median and range. Pain score was compared between the two groups using Mann-Whitney U test. Friedman test was performed to test the significance between the 4-time periods within each group. Chi square test was used to compare qualitative pain score. The level of significance was set at P < 0.05. All tests were two tailed.

## Results

In total, 36 patients were included in this study; Table 1 shows their baseline characteristics. A CONSORT flow diagram can be found in Supplementary File 2.

**Table 1.** Mean and standard deviation of patients’ age and gender enrolled in both groups. ns=non-significant.

Age		
	CH group	DAP group
**Mean**	39.94	33.72
**SD**	13.24	12.72
**t-test**	1.44	
**P-value**	0.1598 ^ns^	
**Gender**		
**Male, n (%)**	2 (11.1)	8 (44.4)
**Female, n (%)**	16 (88.9)	10 (55.6)
**X ^2^ test**	3.462	
**P-value**	0.062 ^ns^	

The overall highest incidence of pain values in both groups was at 6 hours postoperatively, whereas the lowest incidence of pain values was at 48 hours postoperatively with no statistically significant difference between them. The DAP group showed lower postoperative pain values at 12 and 24 hours compared with the CH group, but with no statistical significance difference ( Table 2).

**Table 2.** Median and range values of pain intensity at predetermined time intervals in both groups. ns = not significant.

Pain		Preoperative pain	Postoperative pain (hours)				
Groups			*6*	*12*	*24*	*48*
**Calcium** **hydroxide** **paste**	**Median**	1	3	2	1	0
	**Min**	0	0	0	0	0
	**Max**	10	9	7	9	9
	**Mean rank**	2.89	4.14	3.39	2.50	2.08
**Double** **antibiotic** **paste**	**Median**	2	3	1	0.5	0
	**Min**	0	0	0	0	0
	**Max**	6	5	5	8	10
	**Mean rank**	3.58	4.06	2.78	2.44	2.14
**P-value** (P<0.05)		0.403 ^ns^	0.395 ^ns^	0.053 ^ns^	0.510 ^ns^	0.435 ^ns^

Datasheet containing patients age, gender, and pain score for all time points for both the CH and DAP groups Click here for additional data file. Copyright: © 2018 Samir Abouelenien S et al. 2018 Data associated with the article are available under the terms of the Creative Commons Zero "No rights reserved" data waiver (CC0 1.0 Public domain dedication).

## Discussion

Effective control over the intra-canal microbial load before root canal obturation is a key element that increases the success rate of endodontic treatment ^12^. Necrotic pulp with periapical periodontitis was selected for assessment of postoperative pain, as the highest frequency of postoperative pain occurred in patients with necrotic pulp and apical periodontitis ^4, 13^ that harbored large number of bacteria due to its polymicobial nature as reported in previous studies ^14– 18^. In the present study, pain was recorded using a numerical rating scale (NRS), which is considered a consensus-based, standardized assessment method and reports better compliance compared to other scales ^19^. The literature shows that the NRS provides sufficient distinctive power for chronic pain patients for describing their pain intensity ^20^. As well, it was preferred by the majority of patients in different regions ^21, 22^. In the present study, the Rotary Race system was chosen for root canal preparation, owing to the fact that rotary NiTi instruments result in minimal debris extrusion compared to hand instrumentation ^23^, which in turn would result in less postoperative pain. This was attributed to their rotary action, as well as copious irrigation associated with these instruments ^24^.

In the present study, in patients with necrotic teeth with apical periodontitis, the use of double antibiotic paste showed no difference regarding postoperative pain compared to CH. DAP showed lesser postoperative pain at 12 and 24 hours than the CH group but this didn’t reach a level of statistical significance. This might be attributed to the combined spectrum of antimicrobial activity and synergetic or additive action of antibiotics found in DAP. In accordance with the results of this study, Pai *et al.* ^25^ reported no statistically significant difference in postoperative pain between calcium hydroxide and triple antibiotic paste (TAP), although TAP was more effective that calcium hydroxide but this was not statistically insignificant. As reported in other studies ^7– 9, 26, 27^, there was no statistically significant difference in postoperative pain between calcium hydroxide paste and no intra-canal medication. In contrast, some studies counteracted our findings ^28, 29^, suggesting that CH had pain-preventive properties because of its antimicrobial as well as its tissue-altering effects. These properties could be referred to the chemical, physical, and antimicrobial effects of calcium hydroxide due to the diffusion of its hydroxyl (OH−) ions produced from ionization in aqueous solution, which leads to a highly alkaline environment. This is not conducive for the survival of microorganisms inside root canals ^3, 30^, and most of the microorganisms cannot sustain that high a pH (15.5).

## Conclusion

The use of intra-canal medication for 7 days in necrotic teeth with apical periodontitis was efficient in reducing postoperative pain regardless of the type of intra-canal medication used.

## Data availability

The data referenced by this article are under copyright with the following copyright statement: Copyright: © 2018 Samir Abouelenien S et al.

Data associated with the article are available under the terms of the Creative Commons Zero "No rights reserved" data waiver (CC0 1.0 Public domain dedication). 

F1000Research: Dataset 1. Datasheet containing patients age, gender, and pain score for all time points for both the CH and DAP groups., https://doi.org/10.5256/f1000research.16820.d224359 ^31^

## Supplementary material

Supplementary File 1: CONSORT checklist.

Supplementary File 2: CONSORT flow diagram.

Supplementary File 3: Trial protocol.
