# Supplementary figures and images for: Evaluation of postoperative pain in infected root canals after using double antibiotic paste versus calcium hydroxide as intra-canal medication: A randomized controlled trial
Source: F1000Res. 2018 Nov 8;7:1768. [Version 1] doi: 10.12688/f1000research.16820.1 (PMC6347033; doi:10.12688/f1000research.16820.1)

## Participant flow diagram

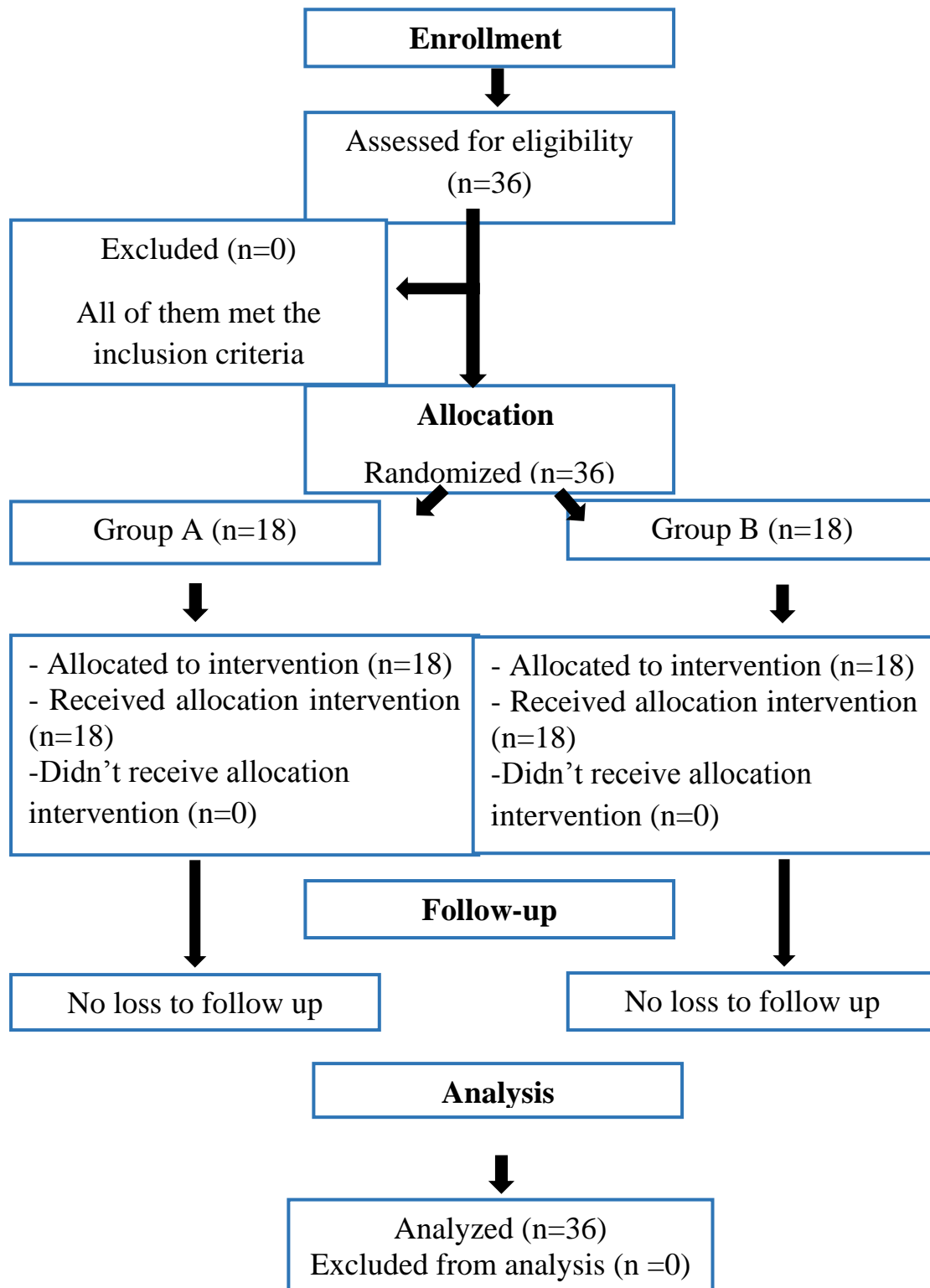

Supplement: Supplementary file 3 [file f1000research-7-18387-s0002.tgz › 9172fb31-8c23-421f-9b14-529f4452d649_Participant_flow_diagram.pdf]
