# Supplementary material for: Evaluation of postoperative pain in infected root canals after using double antibiotic paste versus calcium hydroxide as intra-canal medication: A randomized controlled trial
Source: F1000Res. 2018 Nov 8;7:1768. [Version 1] doi: 10.12688/f1000research.16820.1 (PMC6347033; doi:10.12688/f1000research.16820.1)
Supplement: Supplementary file 4 [file f1000research-7-18387-s0003.tgz › 5e4a886e-548a-486b-b6a3-2616090c6194_Final_protocol.docx]

**Evaluation of postoperative pain and quantification of endotoxins in infected root canals using antibiotic paste versus calcium hydroxide as intra-canal medication**

**(Randomized Clinical Trial)**

**تقييم الالم المصاحب لما بعدعلاج الجذور والتحديد الكمي للسموم الداخلية في قنوات الجذور المصابة باستخدام معجون المضادات الحيوية مقابل هيدروكسيد الكالسيوم كدواء داخل القناه (تجربة إكلينيكية بالانتقاء العشوائي)**

**Protocol**

Submitted to the Faculty of Oral and Dental Medicine-Cairo University in partial fulfillment of the requirement for the Protocol for Doctorate Degree in Endodontics

BY

**Sarah Samir Abouelenien**

BDS, MSc (Cairo University)

Assistant Lecturer of Endodontics

Faculty of Oral and Dental Medicine, Cairo University

(2015)

- **Roles and responsibilities:**

**Investigator**: Sarah Samir

Doctorate degree student

Department of Endodontics

Faculty of Oral and Dental Medicine

Cairo University

**Chief supervisor:** Salsabyl Ibrahim

Professor of Endodontics

Faculty of Oral and Dental Medicine,

Cairo University

- **Funding:** Self-funding.
- **Trial Registration:** To be later online registered on PACTR website.

**Introduction**

Pain is the most common cause for physician consultation. It is the main symptom in many medical and dental conditions and can significantly alter the person's quality of life and general functioning. Endodontic post-treatment pain remains to be significant problem facing the dental profession. The presence of microorganisms as a result of failure to properly disinfect the canal is the most common cause of pain. Thus, endodontic therapy is primarily focused on maximum elimination of these bacteria. Antibacterial intra-canal dressing is advocated to eliminate remaining bacteria after chemo-mechanical preparation.

Calcium hydroxide, the most commonly used intra-canal medication is considered as the most favorable anti-microbial agent (Ghoddusi et al, 2006). It has been proven to be effective against endotoxins as demonstrated by *in-vitro* (Maekawa et al 2011) and *in-vivo* studies (Silva et al 2002). Calcium hydroxide also alters bacterial cell wall and denatures a potent endotoxin, lipopolysaccharide, thereby rendering it less antigenic (Walton et al, 2003).

Lipopolysaccharide (LPS), generally referred to as endotoxins, is one of the most important virulent factors participating in the development and maintenance of apical periodontitis (Pitts et al 1982) (Xavier et al 2013). Lipopolysaccharide is the major constituent of the outer cell wall of gram negative bacteria, which is released during disintegration, multiplication or death of the bacteria (Nair 2004). Clinical investigations have revealed the presence of endotoxins in 100% of the root canal samples in primary and secondary infectious diseases showing apical periodontitis, with high levels related to the development of clinical symptoms (Martinho & Gomes 2008). Because of the high toxicity of endotoxins *in-vivo* and *in-vitro*, even at a very low concentration, its neutralization/removal during endodontic treatment seems to be important for the healing process of the periapical tissues of infected root canals.

Even though calcium hydroxide is one of the most widely used intra-canal medicament due to its anti-microbial properties, there is no clear evidence of its effect on the post-treatment pain after the chemo-mechanical root canal preparation (Anjaneyulu et al, 2014). Other studies found that, Calcium hydroxide was not very effective in reducing post-treatment pain when it was used alone (Ehrmann et al, 2003) (Singh et al, 2013).

Antibiotic paste, is a mixture of metronidazole and ciprofloxacin, which is used as intra-canal medication for disinfection of necrotic teeth. Antibiotic paste has been reported to be effective at reducing bacterial numbers in the root canal systems of infected teeth (Mohammadi & Abbott 2009). When most commonly used medicaments fail in eliminating the symptoms, then antibiotic paste could be used clinically in the treatment of teeth with large periradicular lesions (Taneja & Kumari 2012).

Objectives of the present study: To test the null hypothesis whether the use of antibiotic paste will be more effective in reduction of pain and endotoxins than calcium hydroxide, or will these paste have disadvantages and complains more than the traditional method if used.

# Trial Design:

# Type: Parallel randomized clinical trial

# Allocation ratio: 1:1

# Framework: Superiority

# Aim of the Study:

To assess the ability of antibiotic paste versus calcium hydroxide used as intra-canal medication in reducing pain and level of endotoxins in necrotic teeth with apical periodontitis.

- **PICO approach**

**P:** **population**, patients with non-vital permanent teeth with apical periodontitis.

**I: intervention,** antibiotic paste intra-canal medication.

**C: control,** calcium hydroxide intra-canal medication.

**O: outcomes:**

|  | **Outcome measure** | **Tool for measurement** | **Unit of measurement** |
| --- | --- | --- | --- |
| Primary | Postoperative Pain | Numerical rating scale (Brunelli et al, 2010) | Ordinal |
| Secondary | Quantification of endotoxins | LAL assay | EU/mL |

Postoperative pain will be measured using Numerical Rating Scale (NRS) after 6, 12, 24 and 48 hours from the end of root canal obturation **(Evaristo et al. 2013)**

# Research question:

In patients with necrotic teeth and apical periodontitis would the use of antibiotic paste be more effective in reduction of pain and endotoxins than calcium hydroxide or not?

- **Subjects and Methods:**

**1. Setting and location:**

*** Source of patients**: Out patients of the clinic of endodontic at the faculty of oral and dental medicine, Cairo University, Urban area, Cairo governorate.

*** Operator Qualification:** Post Graduate Master’s Degree Student.

*** Dental units**: Adec 200 U.S.A.

* **X-ray machine**: ViVi, S.r.I, Italy.

* **X-ray film**: Intraoral digital sensor.

1. **Eligibility criteria :**
2. *Inclusion criteria:*
   1. Age between 18-55 years old.
   2. Males or Females.
   3. Maxillary or mandibular single rooted premolar teeth with:

- Non-Vital response of pulp tissue.
- Slight widening in lamina dura.
- Tenderness to percussion.
  1. Positive patients’ acceptance for participation in the study.

1. *Exclusion Criteria*:
   1. Pregnant females: Pain degree change may be due to hormonal change, and some medications are contra indicated.
   2. Patients who had received antibiotic treatment during the last 3 months.
   3. Patients having more than one tooth require root canal treatment.
   4. Teeth with:

- Periodontal probing depth > 4mm.

- Could not be isolated with a rubber dam.

- Fluctuant facial swelling (acute abscess)

- Previous root canal treatment.

- **Final Diagnosis:**
- Mandibular or maxillary single rooted premolar teeth with or without pain.
- Tenderness to percussion.
- On using electric pulp tester it gives response of non-vital pulp.
- On radiographic examination it might have slight widening in lamina dura.
- **Intervention:**

Full medical and dental history using a schematic dental chart will be obtained from all patients treated during this study. Each tooth will be evaluated for vitality (sensitivity) of pulp tissues using electric pulp tester

The patients will be randomly divided into 2 groups:

- ***Experimental group***: antibiotic paste intra-canal medication.
- ***Control group:*** calcium hydroxide intra-canal medication.
- **Sequence of Procedural steps:-**

1. Each patient will be given pain scale chart in order to record his /her pain level before any intervention.
2. Tooth will be anaesthetized using nerve block or infiltration technique by local anesthesia^[[1]](#footnote-1)^.
3. Isolation & disinfection:-

Teeth will be isolated with a rubber dam, and disinfection of their external surfaces will be performed by using 30% hydrogen peroxide followed by 2.5% sodium hypochlorite (NaOCl). Subsequently, 5% sodium thiosulfate will be used to inactivate the disinfectant agents.

1. Access:-

A 2-stage access cavity preparation will be performed. The first stage involved the removal of caries without exposure of the pulp chamber by using a high-speed diamond bur. In the second stage, before entering the pulp chamber, the access cavity will be disinfected according to the decontamination protocol described previously. A new bur will be used to access the canal.

1. 1^st^ sample:-

The first endotoxin sample will be taken by introducing the paper points^[[2]](#footnote-2)^ into the full length of the canal (determined by radiographs and apex locator^[[3]](#footnote-3)^) and retaining them in position for 60 seconds. Next, the paper points will be placed in a glass tube and stored at -80^0^C for the LAL assay.

1. Preparation:-

-The root canals will be prepared with Gates-Glidden drills^[[4]](#footnote-4)^ sizes 4, 3 and 2 for the coronal part, and rotary Irace instruments^[[5]](#footnote-5)^ using an endodontic motor^[[6]](#footnote-6)^ with adjusted torque and speed according to the manufacturer's instructions. The rotary files will be introduced inside the canal using EDTA gel^[[7]](#footnote-7)^.

-Before the use of each instrument, irrigation with a syringe (27-G needle) Containing 3ml 2.5% sodium hypochlorite^[[8]](#footnote-8)^.

- Subsequently, 5% sodium thiosulfate will be used to inactivate NaOCl. Then, 5ml saline solution to wash the canal.

1. 2^nd^ sample:-

The second endotoxin sample will be taken as previously described just after the irrigation with saline solution.

1. Intracanal medication:-

The canals will be dried with paper points and filled with intracanal medication plugged into the canal by using Lentulo spiral^[[9]](#footnote-9)^ . The access cavities will be properly filled with Cavit^[[10]](#footnote-10)^.

- **Control group:** Ca (OH)_2_ Paste.
- **Experimental group:** antibiotic paste (ciprofloxacin, metronidazole)

1. The patient will be instructed to record his/her pain level on the pain scale chart after 6 h, 12 h, 24 h and 48 h.
2. 3^rd^ sample:-

After 7 days of intracanal medication, the canal will be accessed under rubber dam isolation by using the protocol for disinfection described previously. The medication will be removed, the final endotoxin sample will be taken just after the irrigation with saline solution.

1. Obturation:-

Root canals will be obturated using lateral condensation technique by proper selection of master cone^[[11]](#footnote-11)^ corresponding to the same size as the master apical file, and then a spreader will be used to allow space for auxillary cones, using resin-based root canal sealer^[[12]](#footnote-12)^

- **Outcomes:**
- **Primary Outcomes:**

The pain scale (0-10 scale) consists of a line anchored by two extremes "No pain" and "the worst pain", patients will be asked to choose the mark that represents their level of pain from 0 to 10, Pain level will be assigned as follow:

# 0 reading represents “no pain”

# 3 readings represent “mild pain”

# 4- 6 readings represent “moderate pain”

# 7- 10 readings represent “severe pain”

- **Secondary Outcomes:**

The quantitative limulus amoebocyte lysate (LAL) test was used to measure endotoxin concentrations in the root canals by a commercial standard kit. The LAL assay is highly sensitive for detection of minute concentrations of endotoxin. LAL is an aqueous extract of blood cells (amoebocytes) from the horseshoe crab, Limulus polyphemus, that reacts with bacterial endotoxin or LPS. This reaction is the basis of the LAL test, which is used for the detection and quantification of bacterial endotoxins.

- **Participant timeline:**

1. Patients will be selected from the clinic of endodontic after eligibility.
2. They will be randomized and pretreatment diagnosed and radiographed at the same visit of treatment.
3. Patients will be treated in two visits and will record preoperative and postoperative degree of pain at 6, 12, 24 and 48hrs.
4. Patients will return pain charts to the operator on the second visit.

| **periods** | **Recruitment** | **Diagnosis** | **Periapical radiograph** | **Randomization** | **Follow up for postoperative pain** |
| --- | --- | --- | --- | --- | --- |
| Zero |  |  |  |  |  |
| 6 h |  |  |  |  |  |
| 12 h |  |  |  |  |  |
| 24 h |  |  |  |  |  |
| 48 h & close out |  |  |  |  |  |

- **Sample Size:**

The aim of this study is to compare the ability of antibiotic paste versus calcium hydroxide used as intra-canal medication in reducing pain and level of endotoxins in necrotic teeth with apical periodontitis. Based on previous study by Ehrmann, et al. (2003), a medium effect size of approximately 0.25 is expected. A total sample size of 24 patients will be sufficient to detect an effect size of 0.25, a power of 80%, and a significance level of 5%. This number has been increased to a total sample size of 28, to adjust for using nonparametric tests. The number is again increased to a total sample size of 36 (18 in each of the two groups) to allow for losses of around 25%. Sample size was calculated using G*Power program (University of Düsseldorf, Düsseldorf, Germany)^[[13]](#footnote-13)^ . minimal clinical difference 3.5 and 4.5 at 24 hrs and 48 hrs respectively.

# Recruitment:

Patients are recruited for our clinical trial from the clinic of endodontic at the faculty of oral and dental medicine, Cairo University to meet the target sample size.

- **Randomization:**

1. **Sequence generation :**

A random sequence will be generated by computer software,

(http://www.random.org/).

The table will be kept with the assistant supervisor.

1. **Allocation concealment mechanism :**

- Eight folded numbered papers will be packed in opaque sealed envelopes to be dragged by the patients.
- After instrumentation, operator will open the envelope and use the intra-canal medication assigned to that patient. The opaque envelopes will contain the numbers of each random sequence for both groups (AP for experimental group) and (Ca (OH)_2_ for control group).

# Implementation:

The assistant supervisor or co-investigator is the one who will generate the random sequence, assign the participants to the intervention or control groups and the only one who knows whether A or B represents the intervention or the control group.

**Blinding:**

- The study will be double- blinded which is participants and the assessor.
- Participants will not know which group they will be treated with after they will choose the folded paper inside the opaque envelopes which contain the numbers of each random sequence for both groups.
- Assessor who will assess all results data will not know which group the participants related to (fig.8).
- **Data collection, management, and analysis**
- Baseline Data collection:
- Baseline data will be collected by the operator through a report (Fig.1-5), which comprises of 2 charts (one concerning general health and one concerning the chief complaint related events).
- The report will be anonymous where patients identified by their serial numbers (the first letter of the first and last name and date of birth) only will be registered.
- The full detailed personal data of the patient will be written in a separate sheet having the patient's serial number for further contact with patient, this sheet can be only seen by the operator and the assistant supervisor.
- Outcome data collection: (Fig 6, 7)

Primary outcomes will be collected by the operator through (NRS) which is reliable and valid tool (Brunelli et al, 2010). It is scale consisting of numbers from 0 to 10:

# 0 reading represents “no pain”

# 3 readings represent “mild pain”

# 4- 6 readings represent “moderate pain”

# 7- 10 readings represent “severe pain”

Secondary outcomes will be collected by the operator by commercial standard kit then stored at -80^0^ C so that all samples would be processed at the same time by LAL assay for quantification of bacterial endotoxins.

- **Statistical analysis:**

Data will be analyzed using IBM SPSS advanced statistics (Statistical Package for Social Sciences), version 21 (SPSS Inc., Chicago, IL). The level of endotoxins will be described as mean and standard deviation. The level of pain will be described as median and range or interquartile range, as appropriate. The level of endotoxins will be explored for normality using Kolmogrov-Smirnov test and Shapiro-Wilk test. If the exploration of data reveals that the collected values are normally distributed, a 2-way ANOVA with repeated measures will be performed to compare between the 2 groups and the change with time. If the interaction between the groups and time is found to be significant, a 1-way repeated measures ANOVA will be used to examine significant differences between time periods within each of the two groups. The Student’s t-test will be performed to test the significance between the two groups at each time period. If the exploration of data reveals that the level of endotoxins is not normally distributed and for the analyses of the pain score, Mann-Whitney U test will be performed to test the significance between the 2 groups at each time period. Friedman’s test followed by multiple comparisons test will be performed to test the significance between the 4 time periods within each group. In addition, correction of p-value will be done using Bonferroni adjustment to avoid hyperinflation of type 1 error that arises from multiple comparisons. A p-value less than or equal to 0.05 will be considered statistically significant. All tests will be two tailed. The effect size will be measured using standardized mean difference.

- **Data monitoring:**

There will not be a Data Monitoring Committee for the following reasons:

- Interim analysis is not involved in the trial so periodic inspection of the accumulating outcome data is not required.
- Short duration of the trial.
- Minimal risks of the trial.
- **Harms**

Adverse events are minimal or rare. However, if any harm is seen in the participants either in intervention or control groups they will be recorded and reported at the end of the trial. It will be documented as part of routine monitoring not as outcome. The treatment will be according to the harm:

- Pain: administration of Analgesics.
- Swelling: hot fomentation, mouth rinse with salty warm water, antibiotic will be administrated.
- Allergic reaction: referral for a physician for corticosteroid therapy.
- Auditing

In this trial, auditing will be performed in order to preserve the integrity of the trial, the audit will review the processes of participant enrolment, consent, eligibility, allocation to study groups and adherence to trial interventions and policies to protect participants.

- **Ethics and dissemination**
- **Research ethics approval:**
- This protocol and the template informed consent form will be reviewed by the Ethics Committee of Scientific Research, faculty of oral and dental medicine, Cairo University.
- Each of the protocol, informed consent forms in Arabic and English language, recruitment materials and any modifications will be done later on also will be reviewed and approved by the ethical review Committee.
- **Protocol amendments:**

The ethical committees will notified any administrative changes or modifications of the protocol or modification of registered online protocol.

# Consent:

- The trial will be discussed with patients.
- Patients will be able to informed discussion with the researcher.
- Researcher will obtain written consent from patients willing to participate in the trail.
- All consent form will be in Arabic language.
- **­Confidentiality:**
- The confidentiality of the patients can be obtained by using password protected system to secure all local databases
- In this system a coded file will be used to identify all patients' personal data such as name, age, address, phone number, work address.
- The safe secure storage of all files will be done through storage in locked cabinets with limited access.
- **Declaration of interests:**

No conflict of interests.

# Access to data:

The validity of results from our interventional trial can be verified only by the Project Principal Investigators, trial supervisor and trial assistant supervisor, who will have direct full access to their own site’s complete final data sets.

# Ancillary and post-trial care:

Post-trial care planned, patient will be referred to fixed prosthodontics specialist for crown placement after completion of the root canal treatment.

# Dissemination policy:

We are intending to disseminate the results of the trial in the library of the faculty, in the Egyptian dental journal and in the journal of endodontic.

# Informed consent materials:

Patient informed consent:

Ethics Committee will revise and modify according to their guidelines.

Consent: (Fig 9, 10).

- **References:**

Anjaneyulu, K. & Nivedhitha, M.S., 2014. Influence of calcium hydroxide on the post-treatment pain in Endodontics: A systematic review. *Journal of conservative dentistry : JCD*, 17(3), pp.200–7. Available at: http://www.ncbi.nlm.nih.gov/pubmed/4056387.

Brunelli, C. et al., 2010. Comparison of numerical and verbal rating scales to measure pain exacerbations in patients with chronic cancer pain. *Health and quality of life outcomes*, 8, p.42. Available at: http://www.pubmedcentral.nih.gov/articlerender.fcgi?artid=2868814&tool=pmcentrez&rendertype=abstract.

Ehrmann, E.H., Messer, H.H. & Adams, G.G., 2003. The relationship of intracanal medicaments to postoperative pain in endodontics. *International endodontic journal*, 36(12), pp.868–75. Available at: http://www.ncbi.nlm.nih.gov/pubmed/14641427.

Ghoddusi, J. et al., Flare-ups incidence and severity after using calcium hydroxide as intracanal dressing. *The New York state dental journal*, 72(4), pp.24–8. Available at: http://www.ncbi.nlm.nih.gov/pubmed/16925009.

Maekawa, L.E. et al., 2011. In vitro evaluation of the action of irrigating solutions associated with intracanal medications on Escherichia coli and its endotoxin in root canals. *Journal of applied oral science : revista FOB*, 19(2), pp.106–12. Available at: http://www.ncbi.nlm.nih.gov/pubmed/21552710.

Martinho, F.C. & Gomes, B.P.F. a, 2008. Quantification of endotoxins and cultivable bacteria in root canal infection before and after chemomechanical preparation with 2.5% sodium hypochlorite. *Journal of endodontics*, 34(3), pp.268–72. Available at: http://www.ncbi.nlm.nih.gov/pubmed/18291273.

Mohammadi, Z. & Abbott, P. V, 2009. On the local applications of antibiotics and antibiotic-based agents in endodontics and dental traumatology. *International endodontic journal*, 42(7), pp.555–67. Available at: http://www.ncbi.nlm.nih.gov/pubmed/19467048.

Nair, P.N.R., 2004. Pathogenesis of apical periodontitis and the causes of endodontic failures. *Critical reviews in oral biology and medicine : an official publication of the American Association of Oral Biologists*, 15(6), pp.348–81. Available at: http://www.ncbi.nlm.nih.gov/pubmed/15574679.

Pitts, D.L., Williams, B.L. & Morton, T.H., 1982. Investigation of the role of endotoxin in periapical inflammation. *Journal of endodontics*, 8(1), pp.10–8. Available at: http://www.ncbi.nlm.nih.gov/pubmed/6948902.

Silva, L. et al., 2002. Effect of calcium hydroxide on bacterial endotoxin in vivo. *Journal of endodontics*, 28(2), pp.94–8. Available at: http://www.ncbi.nlm.nih.gov/pubmed/11833697.

Singh, R.D. et al., 2013. Intracanal medications versus placebo in reducing postoperative endodontic pain--a double-blind randomized clinical trial. *Brazilian dental journal*, 24(1), pp.25–9. Available at: http://www.ncbi.nlm.nih.gov/pubmed/23657409.

Taneja, S. & Kumari, M., 2012. Use of triple antibiotic paste in the treatment of large periradicular lesions. *Journal of investigative and clinical dentistry*, 3(1), pp.72–6. Available at: http://www.ncbi.nlm.nih.gov/pubmed/22298525.

Walton, R.E., Holton, I.F. & Michelich, R., 2003. Calcium hydroxide as an intracanal medication: effect on posttreatment pain. *Journal of endodontics*, 29(10), pp.627–9. Available at: http://www.ncbi.nlm.nih.gov/pubmed/14606782.

Xavier, A.C.C. et al., 2013. One-visit versus two-visit root canal treatment: effectiveness in the removal of endotoxins and cultivable bacteria. *Journal of endodontics*, 39(8), pp.959–64. Available at: http://www.ncbi.nlm.nih.gov/pubmed/23880258.

**Figure(1)**

**Appendix**


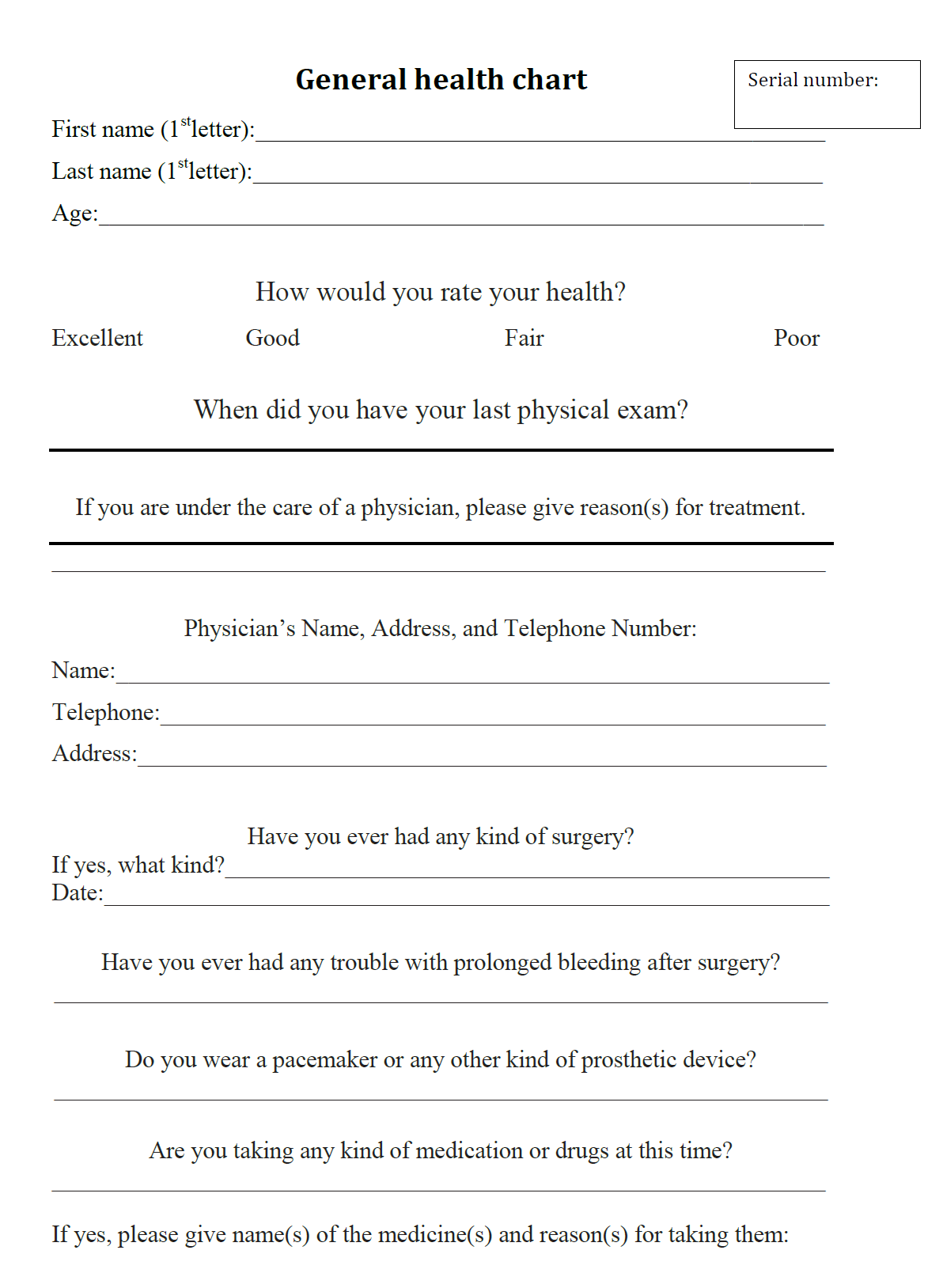


**Figure(2)**


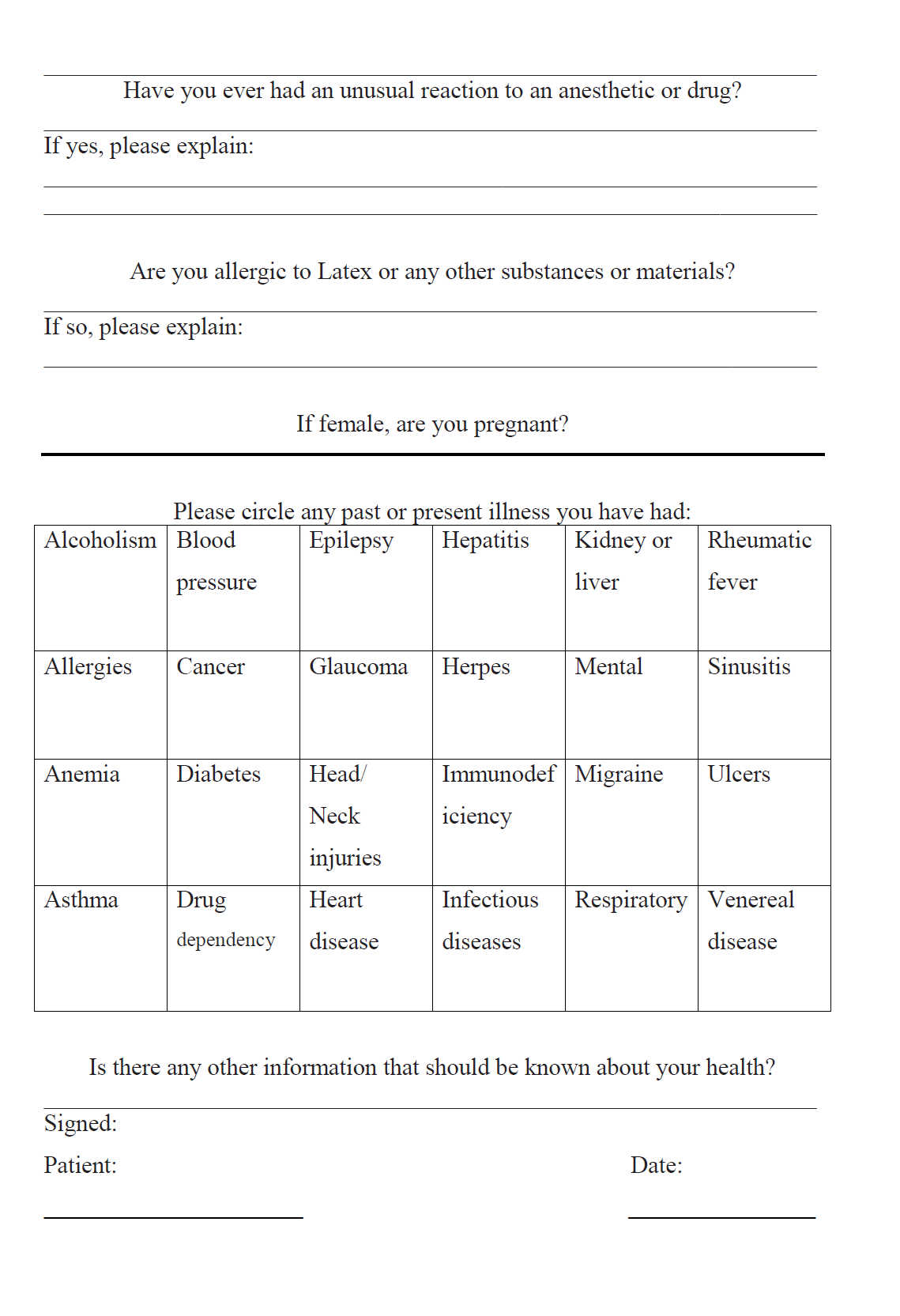


**Figure(3)**


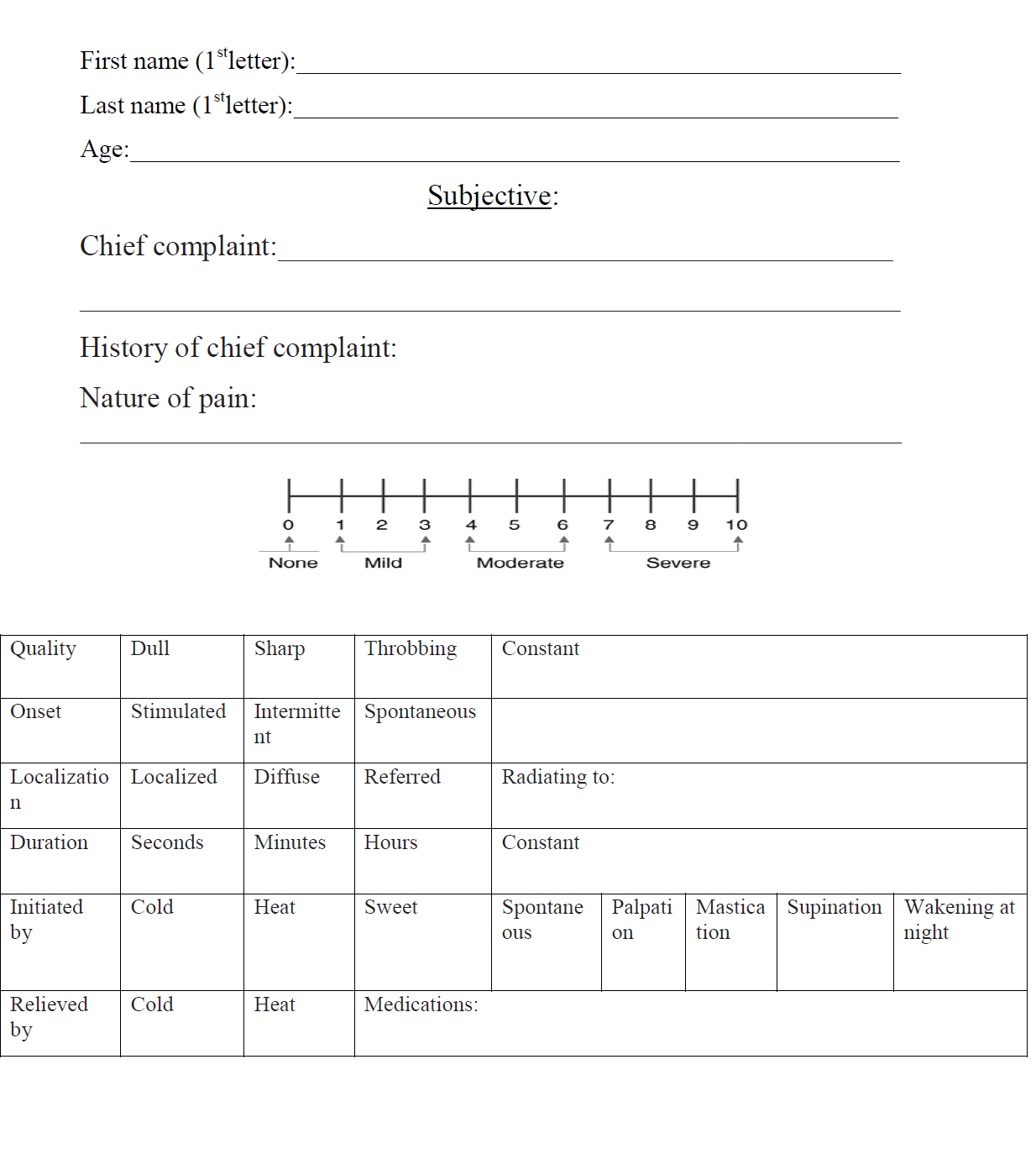

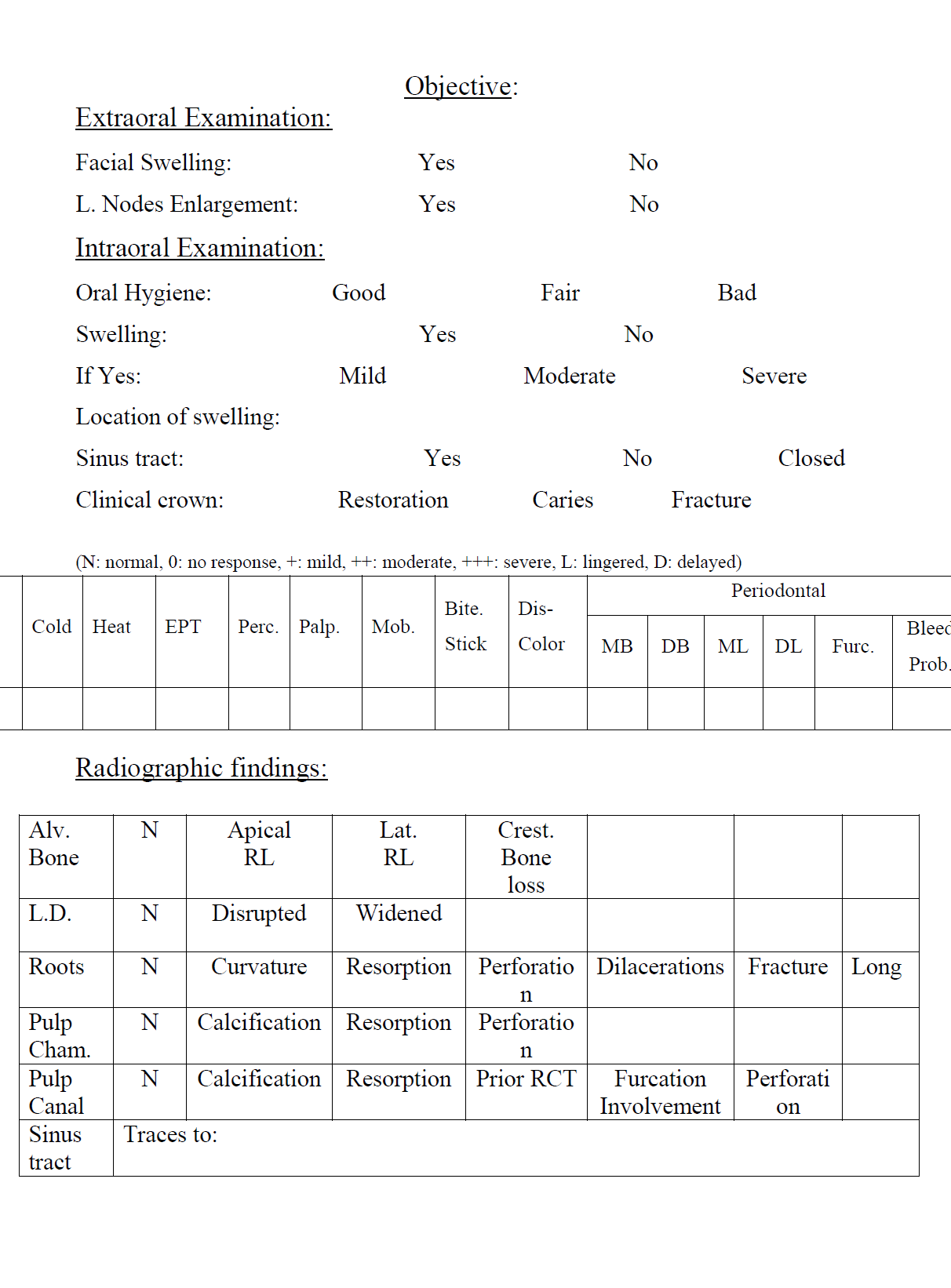


**Figure(4)**


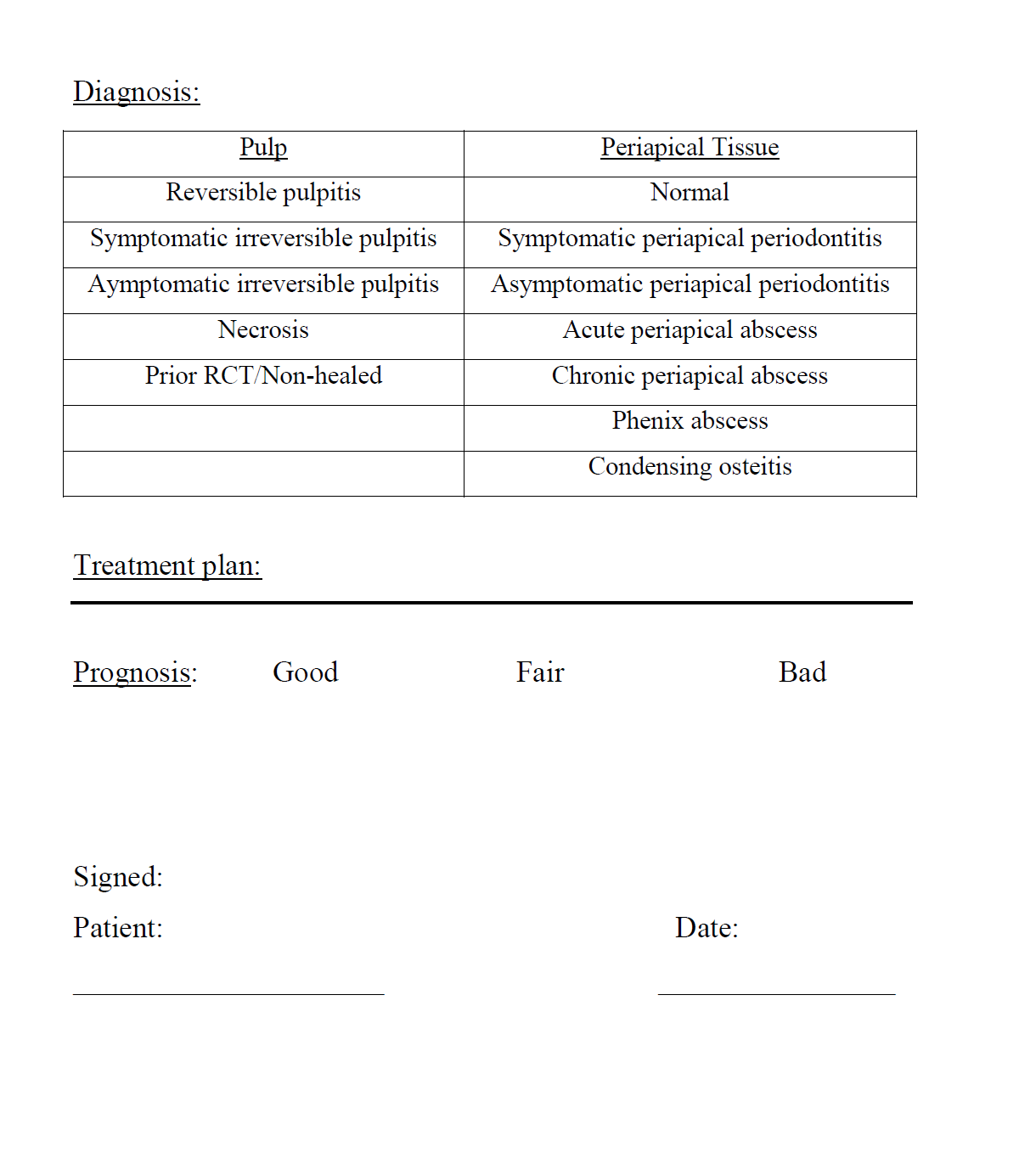


**Figure(5)**

Fig 6

***Numerical Rating Scale (1 _10)***

**اسم المريض: ................................... اسم الطبيب: ........................................**

**رقم المريض: ( ) رقم تليفون الطبيب: ( )**

يرجي وضع علامة علي المقياس ادناة لاظهار شدة ألمك.

1. **Patient training (١) تدريب**


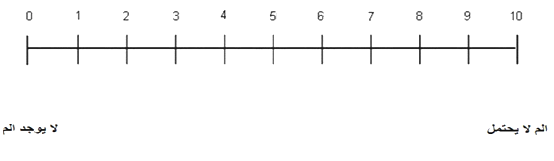


1. **Preoperative (٢) قبل العلاج**


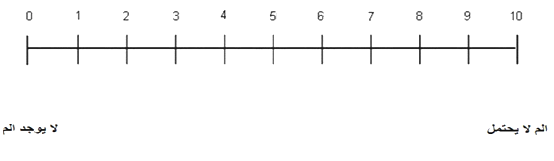


1. **After 6 hours (٣) بعد سته (6) ساعات**


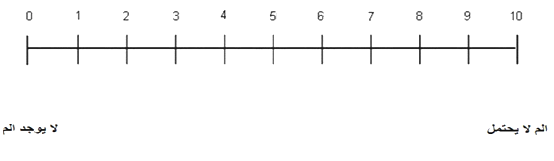


**Figure(6):** pain scale chart

1. **After 12 hours (٤) بعد اثنا عشر (١٢) ساعة**


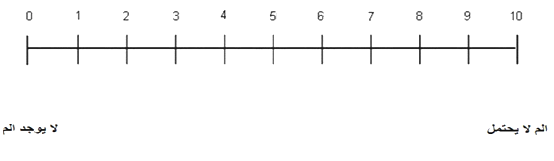


1. **After 24 hours (٥) بعد أربع و عشرون (٢٤) ساعة**


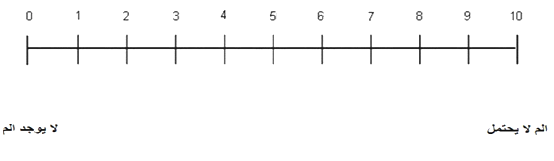


**(٦) بعد ثمان وأربعون (٤٨) ساعة (6) After 48 hours**


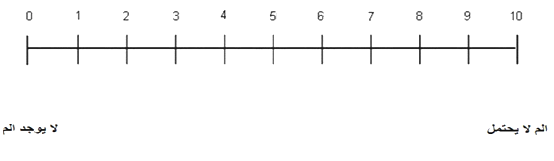


**Figure(7) :** pain scale chart

**Assessor Chart**

**Patient No.:**

| Examiner Assignment | Score | Date | Follow up by hours |
| --- | --- | --- | --- |
|  |  |  | 6 |
|  |  |  | 12 |
|  |  |  | 24 |
|  |  |  | 48 |

**Figure(8) :** The assessor chart

**
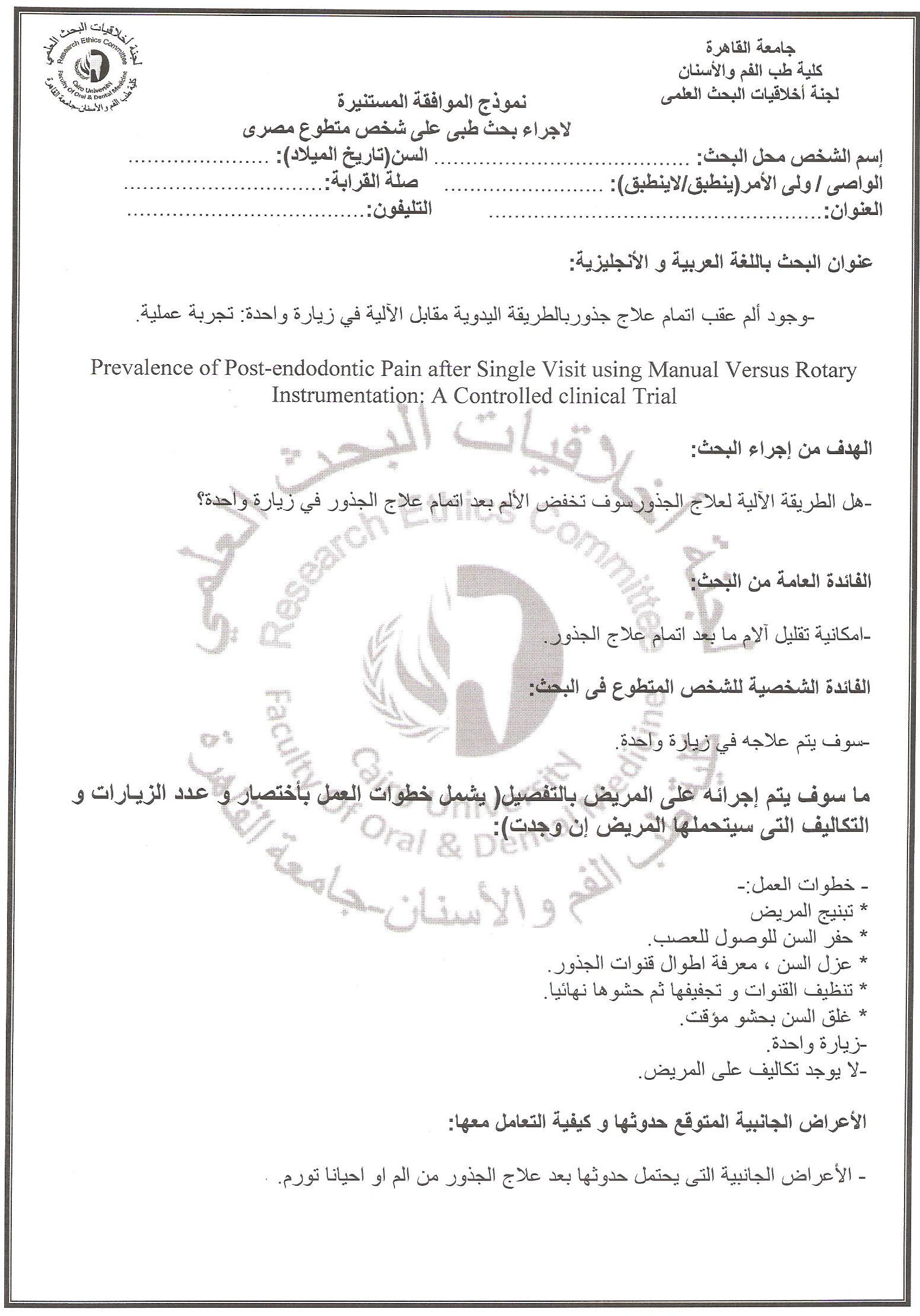
**

**Figure(9) :** The informed consent form given to each patient.


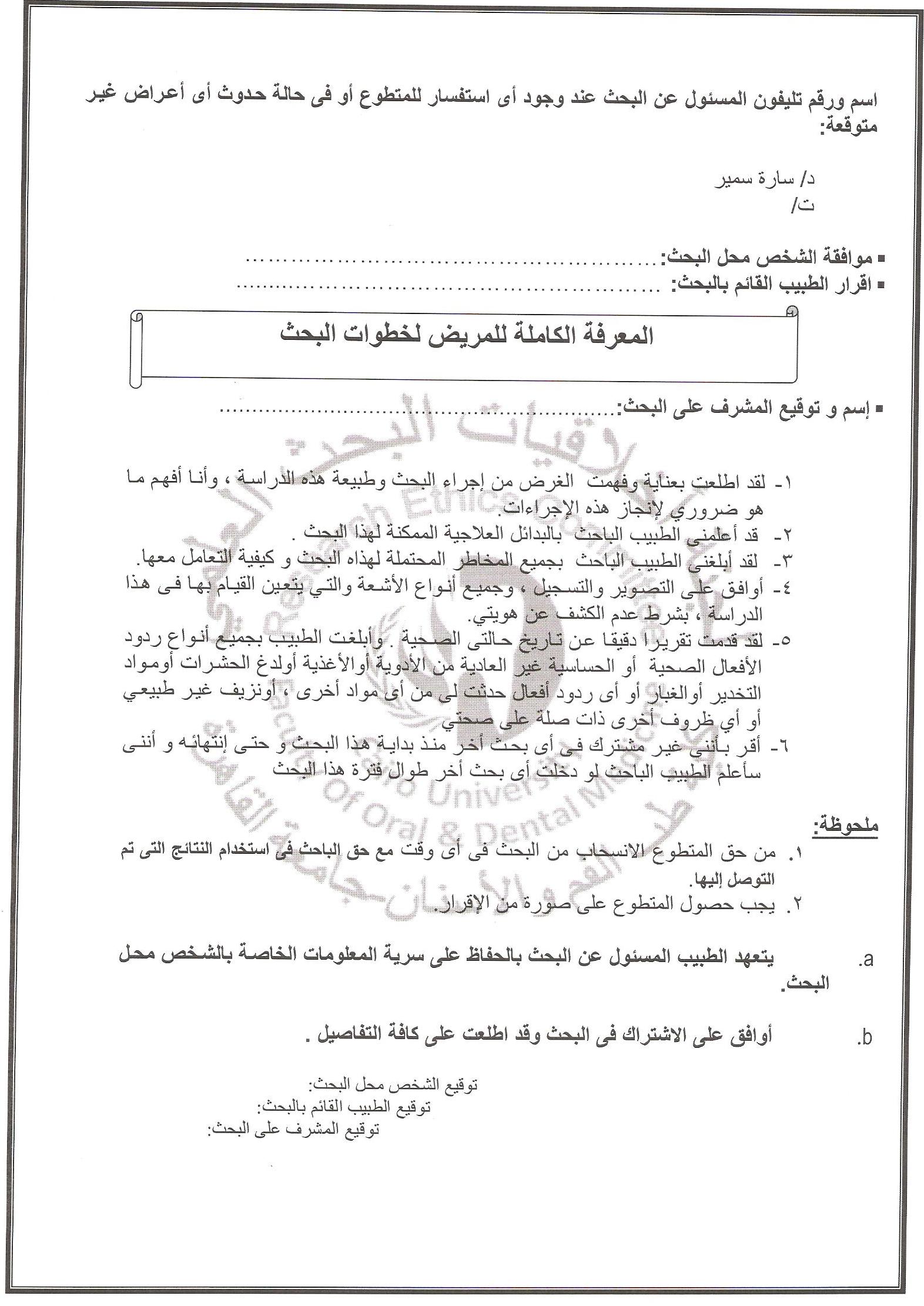


**Figure(10) :** The informed consent form given to each patient.

1. #### Carpule3M™ ESPE™ Ubistesin™ Articaine HCI 4% & Adrenaline 1:100,000 3M Australia

   [↑](#footnote-ref-1)
2. Dentsply Maillefer, Ballaigues, Switzerland [↑](#footnote-ref-2)
3. Root ZX, J.Morita USA, Irvine, CA. [↑](#footnote-ref-3)
4. Mani, Tochigi, Japan [↑](#footnote-ref-4)
5. **FKG Dentaire SA,switzerland.** [↑](#footnote-ref-5)
6. ^5^X-Smart, DentsplyMaillefer, USA.

   6MD-Chelcream, META BIOMED CO.,LTD, Korea.

   7Household Cleaning Products Of Egypt, 10th Of Ramadan, Egypt [↑](#footnote-ref-6)
7. [↑](#footnote-ref-7)
8. . [↑](#footnote-ref-8)
9. Dentsply Maillefer, Ballaigues, Switzerland [↑](#footnote-ref-9)
10. 3M ESPE, Seefeld, Germany [↑](#footnote-ref-10)
11. **FKG Dentaire SA,switzerland** [↑](#footnote-ref-11)
12. ^4^ADSEAL, META BIOMED CO.,LTD, Korea. [↑](#footnote-ref-12)
13. G*Power program (University of Düsseldorf, Düsseldorf, Germany). Faul, F., Erdfelder, E., Lang, A.-G., & Buchner, A. (2007). G*Power 3: A flexible statistical power analysis program for the social, behavioral, and biomedical sciences. Behavior Research Methods, 39, 175-191. [↑](#footnote-ref-13)
