# Supplementary material for: Evaluation of postoperative pain in infected root canals after using double antibiotic paste versus calcium hydroxide as intra-canal medication: A randomized controlled trial
Source: F1000Res. 2018 Nov 8;7:1768. [Version 1] doi: 10.12688/f1000research.16820.1 (PMC6347033; doi:10.12688/f1000research.16820.1)
Supplement: Peer review history

# Peer review: Referee response for version 1

**DOI:** 10.5256/f1000research.18387.r41046

**Published:** 2019-01-24

**Authors:** Brenda P. F.A. Gomes

**Affiliations:** 1 Department of Restorative Dentistry, Division of EndodonticsPiracicaba Dental School , State University of Campinas – UNICAMP, Piracicaba, Brazil

The objective of this study was to assess the ability of double antibiotic paste versus calcium hydroxide used as intra-canal medication in reducing postoperative pain. Post-operative pain is currently a relevant subject in the dental practice. Overall, the study was well written and designed. Additionally, it fits in a Randomized Control Trial, which increases the significance of the research. The results are well described, and no differences were observed between the groups; however, clinically, the antibiotic pastes present some disadvantages compared to calcium hydroxide. The sample size is adequate for this kind of research. I recommend the manuscript for indexing.

I have read this submission. I believe that I have an appropriate level of expertise to confirm that it is of an acceptable scientific standard.

# Peer review: Referee response for version 1

**DOI:** 10.5256/f1000research.18387.r40440

**Published:** 2018-11-12

**Authors:** Reham Hassan

**Affiliations:** 1 Faculty of Dentistry, Department of Endodontics, Minia University, Minya, Egypt

This article addresses the links between intra-canal medication used and the post operative pain after endodontic treatment, as mentioned in the article, post operative pain is multifactorial, however the design of the experiment is adequate including experimental and control groups, the primary outcome was measured preoperatively and after 6, 12, 24 and 48 hours only, however in the conclusion its mentioned that the 7 days was sufficient for reducing the post-operative pain. (The use of intra-canal medication for 7 days in necrotic teeth with apical periodontitis was efficient in reducing postoperative pain regardless of the type of intra-canal medication used). 

- **Approved: **No changes are required.  The experimental design, including controls and methods, is adequate; results are presented accurately and the conclusions are justified and supported by the data.
- **The research is clear and cites the current literature in the topic of post-operative pain after intra-canal medication.**
- The data published and the supplemental data provided is sufficient and clear to allow replication 
- The conclusion is sufficiently supported by the results but could omit the (7 days) to prevent confusion.

I have read this submission. I believe that I have an appropriate level of expertise to confirm that it is of an acceptable scientific standard.
